# DB-YOLO: A Dual-Branch Parallel Industrial Defect Detection Network

**DOI:** 10.3390/s25216614

**Published:** 2025-10-28

**Authors:** Ziling Fan, Yan Zhao, Chaofu Liu, Jinliang Qiu

**Affiliations:** College of Architectural and Engineering, Yunnan Agricultural University, Kunming 650201, China; 2022313430@stu.ynau.edu.cn (Z.F.); 2014043@ynau.edu.cn (J.Q.)

**Keywords:** YOLO, mamba, insulator defect detection

## Abstract

Insulator defect detection in power inspection tasks faces significant challenges due to the large variations in defect sizes and complex backgrounds, which hinder the accurate identification of both small and large defects. To overcome these issues, we propose a novel dual-branch YOLO-based algorithm (DB-YOLO), built upon the YOLOv11 architecture. The model introduces two dedicated branches, each tailored for detecting large and small defects, respectively, thereby enhancing robustness and precision across multiple scales. To further strengthen global feature representation, the Mamba mechanism is integrated, improving the detection of large defects in cluttered scenes. An adaptive weighted CIoU loss function, designed based on defect size, is employed to refine localization during training. Additionally, ShuffleNetV2 is embedded as a lightweight backbone to boost inference speed without compromising accuracy. We evaluate DB-YOLO on the following three datasets: the open source CPLID, a self-built insulator defect dataset, and GC-10. Experimental results demonstrate that DB-YOLO achieves superior performance in both accuracy and real-time efficiency compared to existing state-of-the-art methods. These findings suggest that the proposed approach offers strong potential for practical deployment in real-world power inspection applications.

## 1. Introduction

In recent years, the need for a reliable and safe power supply has grown rapidly with societal development. Insulators, as essential components of power transmission systems, are critical for maintaining line stability and safety. However, prolonged exposure to harsh outdoor conditions—such as extreme temperatures, intense UV radiation, and environmental pollution—can cause various defects, including cracks, breakages, deformations, and contamination. Such defects may compromise both electrical insulation and mechanical integrity, potentially resulting in flashovers, line trips, or large-scale power outages. Consequently, the accurate and efficient detection of insulator defects is of paramount importance [1].

Recent studies have increasingly focused on small-scale defects, also known as incipient faults, which are subtle yet often precede severe failures. Detecting such early-stage defects requires sensitive and robust methods. Some data-driven techniques have been explored in some engineering fields to capture the weak signals of incipient faults [2,3,4]. However, these methods are primarily designed for mechanical or control systems using residual analysis, and they cannot be directly applied to image-based insulator defect detection. The key distinction of this work is that we propose a vision-based deep learning approach specifically tailored to detect fine defects in insulator images. By incorporating the concept of early-stage sensitivity into the network design, our method achieves high-precision recognition of small-scale defects. Compared with traditional approaches and existing residual-based methods, our approach is better suited to handle complex backgrounds and diverse defect patterns in visual data.

Currently, insulator defect detection methods can be broadly divided into traditional image processing- and deep learning-based approaches. Traditional techniques usually rely on handcrafted features combined with classical classifiers such as Support Vector Machines (SVMs) [5] or Random Forests [6]. For example, Guo et al. [7] proposed an automated detection method based on Principal Component Analysis (PCA), while Luo [8] combined SVM with Quantum Particle Swarm Optimization (SVM-QPSO) to enhance classification accuracy.

However, due to the complex backgrounds and diverse shapes of defects in insulator images, it is difficult to extract effective features using handcrafted methods. This complexity limits the performance of traditional recognition algorithms and often leads to low detection accuracy. In contrast, deep learning methods, especially convolutional neural networks (CNNs) [9], can directly learn hierarchical features from raw images, enabling end-to-end defect detection without the need for manual feature design. Compared with traditional approaches, deep learning offers better accuracy, faster inference, and improved generalization. Ferguson et al. [10] explored the effectiveness of feature extractors like VGG-16 and ResNet-101 in defect detection tasks. Xu et al. [11] enhanced defect detection performance by leveraging feature cascades within VGG-16. Li et al. [1] proposed a Two-Stage Defect Detection Framework based on Improved-YOLOv5 and Optimized-Inception-ResNetV2, which improves the accuracy of small defect localization through architectural enhancements and attention mechanisms.

Despite these advancements, deep learning-based models still face challenges in detecting small-scale or irregularly shaped insulator defects, particularly when real-time performance is required in practical applications. Many existing networks are computationally intensive and structurally redundant, making them unsuitable for deployment on lightweight edge devices such as drones used in power line inspections. Moreover, the variation in defect sizes and patterns further complicates detection. To overcome these limitations, researchers have explored multi-branch and multi-scale YOLO models that explicitly enhance feature representation across different object sizes. For example, MAF-YOLO [12] integrates multi-branch auxiliary fusion to improve small-object perception, UAV-YOLOv8 [13] rethinks multi-scale representation learning to achieve a better balance between accuracy and speed, and RDS-YOLO [14] introduces multi-scale feature fusion tailored for dense small targets. These studies highlight the importance of designing detection frameworks that adapt to varying target scales while ensuring computational efficiency.

In this study, we aim to address these challenges by introducing a dual-branch object detection framework tailored for insulator defect detection. Our method is designed to accurately recognize defects of different sizes and appearances by explicitly incorporating a large-defect detection branch and a small-defect detection branch, thus enhancing robustness across scales. At the same time, we optimize the architecture to maintain real-time performance suitable for aerial inspection scenarios using unmanned aerial vehicles (UAVs), ensuring both detection accuracy and deployment feasibility.

To address the challenges of detecting insulator defects with varying sizes, this paper introduces an enhanced detection algorithm based on YOLOv11, incorporating a dual-branch architecture. The model is designed with the following two specialized detection branches: one focusing on large-scale defects and the other on small-scale defects. This targeted approach allows the network to more effectively handle multi-scale defect features, significantly improving overall detection accuracy without compromising the lightweight and efficient nature of the original YOLO framework. To further enhance global perception, we incorporate the Mamba mechanism, which improves contextual feature extraction, particularly in cluttered scenes where large defects may be missed. Additionally, we introduce an adaptive weighted CIoU loss that dynamically adjusts parameters based on defect size, promoting better convergence during training and enabling precise localization of both small and large defects. This approach enhances model robustness and accuracy while reducing training complexity. Compared with existing state-of-the-art methods, the proposed network delivers higher detection accuracy without compromising inference speed, demonstrating strong potential for real-time insulator inspection and large-scale industrial applications.

1. This study presents a dual-branch insulator defect detection method built on YOLOv11, which enhances detection accuracy while retaining the model’s lightweight characteristics. The approach adopts a dual-branch design, comprising separate branches for large and small defects, allowing the network to specialize in detecting targets of varying sizes.
2. To better detect large-scale defects, the Mamba algorithm is incorporated to strengthen the network’s global feature extraction. By capturing more comprehensive contextual information, the Mamba mechanism enables the accurate identification of extensive defect regions, even in complex scenes with cluttered backgrounds and local interference.
3. To optimize detection performance across various defect scales, an adaptive weighted CIoU loss function is designed. The loss parameters are dynamically adjusted based on the size of the defect, which facilitates model convergence during training and enhances detection accuracy for both large and small defects.
4. Compared with current state-of-the-art detection models, the proposed network delivers superior accuracy while sustaining high inference speed, making it well-suited for real-time insulator inspection and practical industrial deployment.

## 2. Related Works

### 2.1. Object Detection

Over the past decades, numerous strategies for object detection have been proposed from different perspectives. Early approaches mainly relied on handcrafted feature design [15], often combined with classifiers such as SVMs [5] for recognition. With the rise of deep learning in computer vision, fully convolutional network (FCN)-based detection frameworks [16] have become mainstream, and these methods are generally categorized into two-stage and one-stage paradigms [17].

Two-stage detection is exemplified by the Faster R-CNN family [18], where the first stage generates feature representations and regions of interest (ROIs), followed by classification and regression in the second stage. Although this architecture achieves high accuracy, it suffers from considerable inference latency.

In contrast, one-stage detectors, such as the YOLO series [19], directly divide the feature map into grids, each responsible for predicting object categories and bounding boxes. Anchor-based detectors including RetinaNet [20] and EfficientDet [21] follow a similar design principle. Owing to their efficiency, one-stage methods are widely applied in industrial defect detection tasks. The one-stage object detection network consists of the following three parts: backbone, neck, and detector. The backbone is responsible for feature extraction. The neck is used to fuse features. The detector is used for object detection. We designed a dual-branch YOLO algorithm based on YOLOv11, which takes advantage of YOLO’s lightweight architecture while significantly enhancing detection accuracy. The dual-branch structure allows the model to specialize in detecting different types of targets—one branch focuses on large targets and the other on small targets. This specialization enables the model to better handle a wider range of object sizes, ensuring more accurate detections. By combining the efficiency of YOLO’s lightweight design with a more focused detection strategy through the dual-branch approach, we were able to strike a balance between speed and precision, making the algorithm both fast and highly accurate.

### 2.2. Vision in Mamba

Based on the study of SSM [22], Mamba [23] exhibits linear complexity in input size, addressing the computational efficiency issue of the Transformer on long sequences of modeling state space.

Within the development of general-purpose visual backbones, Vision Mamba [24] introduced the first pure vision-oriented backbone based on selective SSM, marking the initial application of Mamba in computer vision. Building on this, VMamba [25] proposed the Cross-Scan mechanism, enabling 2D selective scanning to enhance visual representation and achieving strong results in image classification. LocalMamba [26] adopts a window-based scanning strategy, refining local dependency modeling while incorporating dynamic scanning to adaptively select optimal configurations across layers. To alleviate the heavy computational cost of self-attention in Transformer-based detectors, Mamba YOLO integrates an ODMamba backbone with a State Space Model (SSM), thereby reducing complexity [27].

We designed a branch specifically for large targets, leveraging the advantages of the Mamba algorithm to enhance global feature extraction, thereby improving detection accuracy. The Mamba algorithm allows the network to better capture and utilize global contextual information, which is crucial for accurately identifying large targets. By focusing on global features, we are able to ensure that the network not only detects the target but also distinguishes it from the surrounding environment more effectively. This results in a more robust detection performance, especially in complex or cluttered backgrounds where large targets might otherwise be missed.

### 2.3. IoU Loss

The regression loss of bounding boxes plays a pivotal role in determining object localization accuracy. To overcome the limitation of zero gradients when the predicted and ground truth boxes do not overlap, Rezatofighi et al. [28] proposed the GIoU Loss. Building upon this, Zheng et al. [29] introduced the DIoU Loss, which incorporates not only overlap but also center distance and aspect ratio consistency, and later extended it to CIoU Loss. Subsequently, Zhang et al. [30] refined CIoU by replacing aspect ratio consistency with independent penalties on length and width, resulting in the EIoU Loss.

However, prior studies have largely neglected the imbalance in loss across objects of different scales. Such bias can misguide the network’s focus, hindering effective weight updates [31]. To address this, we designed adaptive weighted CIoU loss functions with scale-specific parameters, significantly reducing training difficulty and enhancing accuracy. By dynamically adjusting IoU loss according to object size, the model achieves better precision in detecting both small and large targets. This tailored design improves sensitivity to small objects while maintaining robustness for larger ones, ultimately leading to faster convergence and superior detection performance.

## 3. Materials and Methods

### 3.1. Overview

**Figure 1:**
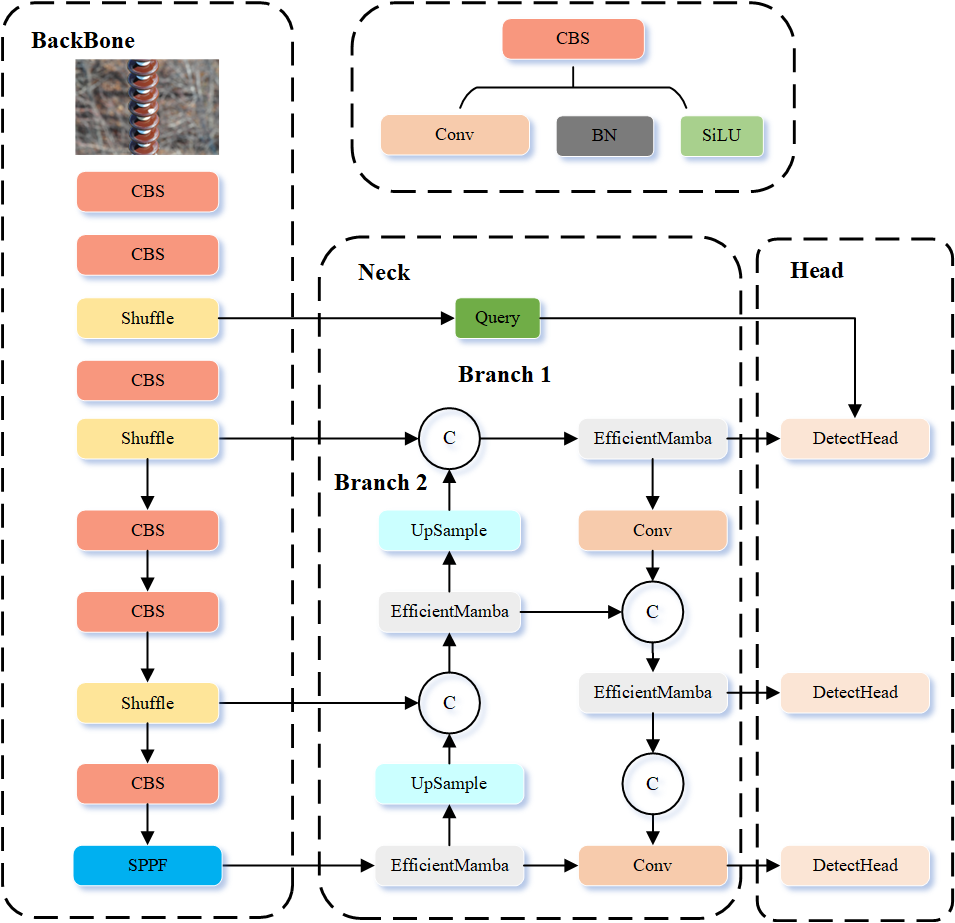
Structure of our method.

The overall framework of the proposed network is shown in Figure 1. In practical insulator defect detection, defect sizes vary significantly, from tiny flaws spanning only a few pixels to large defects covering substantial portions of the image. Such scale variation presents a major challenge for conventional detectors, which often fail to achieve balanced performance across small and large targets. To address this issue, we propose DB-YOLO, a dual-branch YOLO-based model tailored for multi-scale defect detection.

The key idea of DB-YOLO is its dual-branch design, where two specialized branches are optimized for different defect sizes. Specifically, the large-defect branch focuses on wide-area flaws, while the small-defect branch emphasizes fine-grained target recognition. This structure effectively mitigates the scale imbalance problem, yielding improved detection accuracy across diverse defect categories.

To further boost performance, several architectural enhancements are introduced. First, the YOLOv11 backbone is replaced with ShuffleNetV2, a lightweight alternative that reduces parameters and accelerates inference while preserving accuracy, enabling real-time deployment. Second, the large-defect branch integrates the Mamba module, which strengthens global feature extraction and enhances contextual perception. In parallel, the small-defect branch employs the QueryDet mechanism, which improves the detection of small targets by focusing on fine-grained representations. The synergy of these modules enhances both efficiency and detection precision.

In addition, an adaptive weighted CIoU-based loss is designed to tackle optimization difficulties arising from scale variation. By dynamically adjusting according to defect size, the loss function enables balanced optimization for both small and large targets. This adaptive design not only improves detection accuracy but also accelerates model convergence.

The following sections provide detailed discussions of the Efficient Mamba Block, the Cascaded Query Mechanism, and the Adaptive Weighted CIoU Loss.

### 3.2. Efficient Mamba Block

In insulator defect detection, one of the major challenges is the efficient detection of large defects, which often occupy a significant portion of the image. Traditional detection methods often perform poorly when dealing with large-scale defects, as they may fail to capture the broad contextual information necessary for accurate detection. This leads to computational bottlenecks, where the model either sacrifices speed or detection accuracy when processing larger defects.

Traditional State Space Models (SSMs) face limitations in handling large-scale visual tasks due to their global information extraction complexity of $𝒪(N^{2})$, where *N* is the spatial resolution dimension of the input feature map (e.g., H×W).

**Figure 2:**
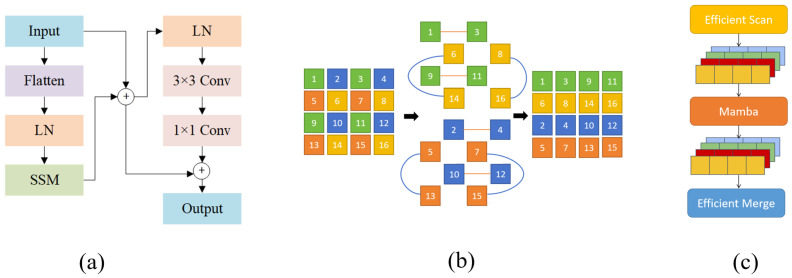
(**a**) Mamba Block. (**b**) Description of EMB. (**c**) Implementation of EMB.

To reduce this computational complexity, EMB introduces a selective scanning strategy that combines dilated convolution with an efficient skipping sampling mechanism. This module is shown in Figure 2, where (a) represents the Mamba Block, (b) illustrates the EMB, and the overall implementation is also depicted. Specifically, EMB divides the input feature map into multiple blocks and performs scanning with a stride *p*, effectively skipping positions in the spatial dimension. Instead of scanning every spatial location, EMB selects sparse key points where the following hold:

(1) imodp=0,jmodp=0

This reduces the number of tokens to be processed and lowers the complexity to $𝒪\left(\frac{N}{p^{2}}\right)$, significantly reducing computational cost while preserving a global receptive field.

After scanning, EMB reconstructs the global structure of the feature map by merging these blocks, enabling the model to capture more comprehensive contextual information—a crucial factor in detecting large-area defects.

Furthermore, the Mamba Block enhances the capability of SSMs through the Selective State Space (S6) mechanism, in which the parameters dynamically adapt to the input. The state update function is defined as follows:

(2) $$h_{t}=A\left(x_{t}\right)\cdot h_{t-1}+B\left(x_{t}\right)$$

Here, $h_{t}\in R^{d}$ is the output vector at step *t*, $h_{t-1}$ is the previous hidden state, and $x_{t}\in R^{d_{x}}$ is the current input feature vector. The transformation matrices $A(x_{t})$ and bias vectors $B(x_{t})$ are functions of the current input, expressed as follows:

(3) $$A\left(x_{t}\right)=f_{A}\left(x_{t};\theta_{A}\right),\;B\left(x_{t}\right)=f_{B}\left(x_{t};\theta_{B}\right)\;$$

where $f_{A}$ and $f_{B}$ denote lightweight parameterized functions (1×1 convolutions) and $\theta_{A},\theta_{B}$ are learnable parameters. This design allows state $h_{t}$ to adapt dynamically to the input context, selectively emphasizing informative features while efficiently propagating information from previous states.

Finally, these features are fed into a Selective State Intermediate Layer, where the extracted representations more effectively reflect relevant information. This enables efficient and accurate large-defect detection without sacrificing real-time performance.

### 3.3. Cascaded Query Mechanism Based on QueryDet

In FPN-based detectors, small objects are typically detected using high-resolution feature maps with weak semantic information. However, such small objects only occupy a small portion of the image, and their localization performance tends to degrade significantly. This issue becomes more pronounced in insulator defect detection tasks, where defects are often sparsely distributed across the image and vary in size. Directly adding a layer dedicated to small-object detection in the network would significantly increase the computational cost, severely affecting real-time processing capabilities and making it difficult to balance accuracy and speed.

**Figure 3:**
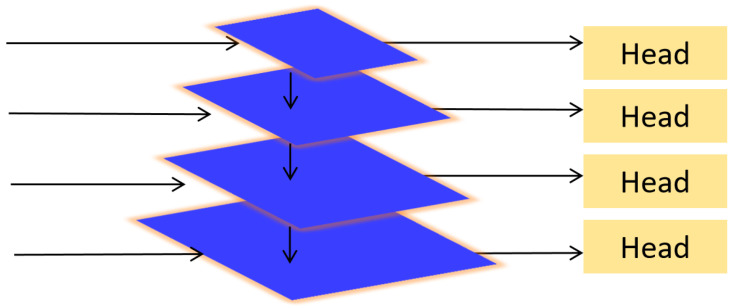
Structure of QueryDet.

To address this, we introduce the QueryDet [32] module in the small-defect detection branch of the proposed DB-YOLO model. For the preprocessed $P_{3}$ and $P_{4}$ layers (as shown in Figure 3), we construct query (Query), key (Key), and value (Value) feature sets, and we perform sparse query operations using attention mechanisms to obtain enhanced feature maps for small object regions. These are then combined with the high-resolution spatial information from the $P_{2}$ layer to accurately detect small objects.

The Query-block converts the feature maps from $P_{3}$ and $P_{4}$ into query-key-value triplets, which are used in subsequent attention operations. The image first passes through the backbone and feature pyramid network to extract multi-scale features, with the highest resolution $P_{2}$ layer chosen as the primary feature layer. Subsequently, the Query-block utilizes high-confidence indexes from $P_{3}$ and $P_{4}$ to perform sparse queries on $P_{2}$ and generate fine-grained feature maps for high-resolution detection.

This encoding strategy is used to generate target maps for small objects and is involved in IoU calculations, non-maximum suppression (NMS), and the optimization of the regression loss function.

**Figure 4:**
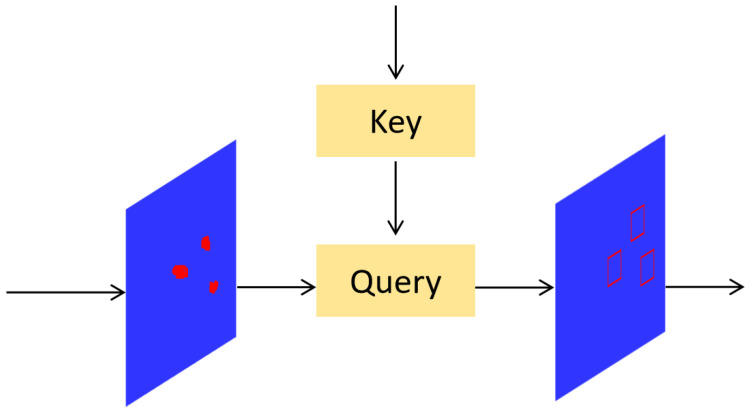
Structures of Query-block. The red regions represent the feature map of the target.

Figure 4 provides a simplified diagram where the left input is the processed fine-grained feature map, and the right input is the key region index map obtained via coarse querying. The query module extracts these high-confidence regions from the fine-grained feature map, and then high-resolution detection is applied to these regions, improving the detection performance for small defects.

Unlike traditional methods that add a separate layer for small-object detection, QueryDet leverages existing multi-scale features and introduces attention mechanisms to intelligently filter key detection regions. This approach improves small-object detection accuracy without adding computational overhead. It effectively enhances small-defect detection capabilities while maintaining high real-time performance.

### 3.4. Adaptive Weighted CIoU Loss

In YOLOv11, the default bounding box regression relies on the standard Intersection over Union (IoU) loss. Although widely used, IoU-based losses often suffer from limitations such as inaccurate localization for highly overlapping boxes and insensitivity to aspect ratio or scale variations. These shortcomings are particularly problematic in industrial defect detection, where object sizes and shapes vary considerably.

To overcome these issues, we replace the standard box loss with an adaptive weighted Complete IoU (CIoU) loss. CIoU [33] extends traditional IoU by incorporating both aspect ratio and center distance, offering a more comprehensive measure for localization accuracy. Beyond this, we further enhance CIoU by introducing an adaptive confidence weighting strategy, which dynamically adjusts confidence penalties according to the detection difficulty of individual objects. This mechanism ensures that the network allocates greater attention to hard-to-detect instances, thereby improving robustness in defect detection.

For single-class detection, the weight adjustment depends on the predicted confidence score as follows: predictions with lower confidence are penalized more heavily, forcing the network to refine uncertain regions. This adaptive design effectively mitigates the challenges posed by scale variation, ensuring balanced optimization across both small and large targets. The formulation of the weighted CIoU loss is presented in Equation (4).

(4) $$L_{AWCIoU}=\left(1-IoU+\frac{p^{2}\left(bp,bg\right)}{c^{2}}+\alpha \varphi \right)\times W_{l}$$

where IoU denotes the intersection over the union between the predicted box $b_{p}$ and ground truth box $b_{g}$, $p^{2}\left(b_{p},b_{g}\right)$ is the squared Euclidean distance between their centers, *c* is the diagonal length of the minimum enclosing box covering both, α and φ preserve aspect ratio consistency, and $W_{l}=\exp\left(-\mathrm{confidence}\right)$ is a size-dependent weight that emphasizes low-confidence predictions. This adaptive design forces the network to refine uncertain regions, effectively mitigating challenges from scale variation and improving the detection of difficult, low-contrast defects while maintaining stability for high-confidence predictions.

## 4. Results

### 4.1. Datasets

To validate the effectiveness of the proposed insulator defect detection algorithm, we conducted experiments separately on two publicly available datasets and one self-constructed dataset from the Lijiang Power Grid in Yunnan Province. The detailed descriptions of the three datasets are provided in Table 1. We briefly introduce the datasets, evaluation metrics, and training setup, and we finally present experimental results with comparisons to existing methods.

**Figure 5:**
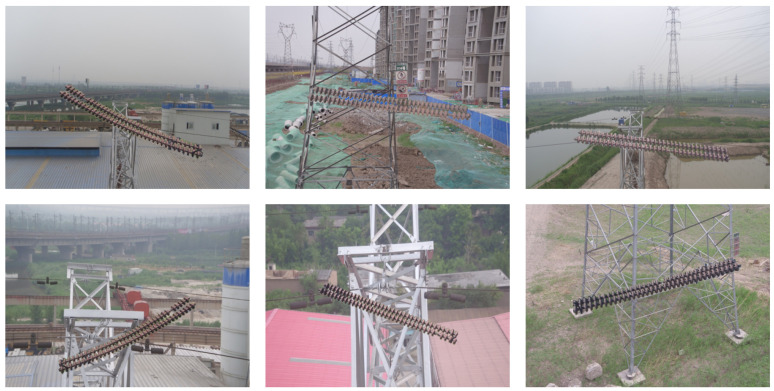
Chinese Power Line Insulator Dataset.

The Chinese Power Line Insulator Dataset (CPLID) [34] consists of 600 real-world images of intact insulators and 248 synthetically generated images of defective ones. All images were acquired using UAVs and are provided by the State Grid Corporation of China. Representative samples are illustrated in Figure 5.

**Figure 6:**
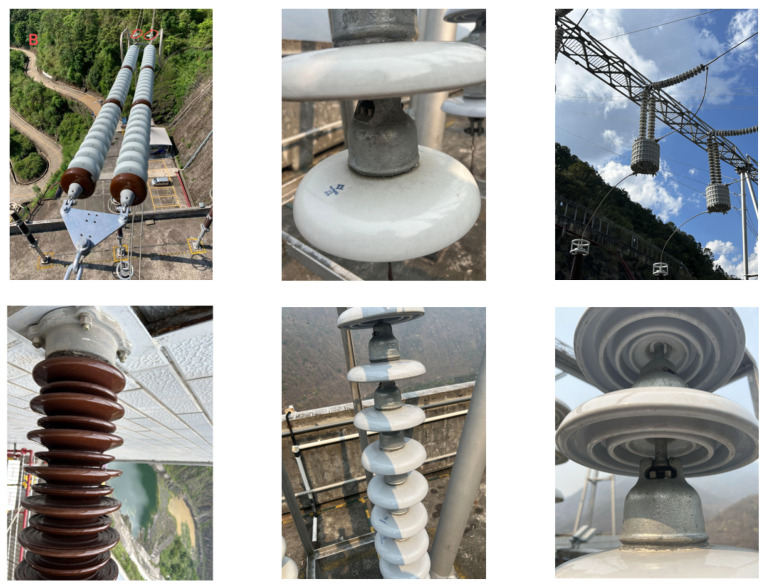
Yunnan Lijiang Power Line Insulator Dataset.

The Lijiang dataset, collected in collaboration with Yunnan Power Grid Co., Ltd., Kunming, China, consists of 4000 high-resolution images taken under real-world conditions using UAV-mounted cameras. It covers diverse scenarios including strong lighting, occlusion, and background interference. It contains six label categories, namely, Crack (Cr), Contamination (Co), Broken (Br), Burn (Bu), Rust (Ru), and Missing Cap (Mc). All defect regions were annotated by experts in YOLO format. The dataset is split into training, validation, and test sets (70%/15%/15%) and augmented with random cropping, flipping, and brightness adjustments. Representative samples are illustrated in Figure 6.

**Figure 7:**
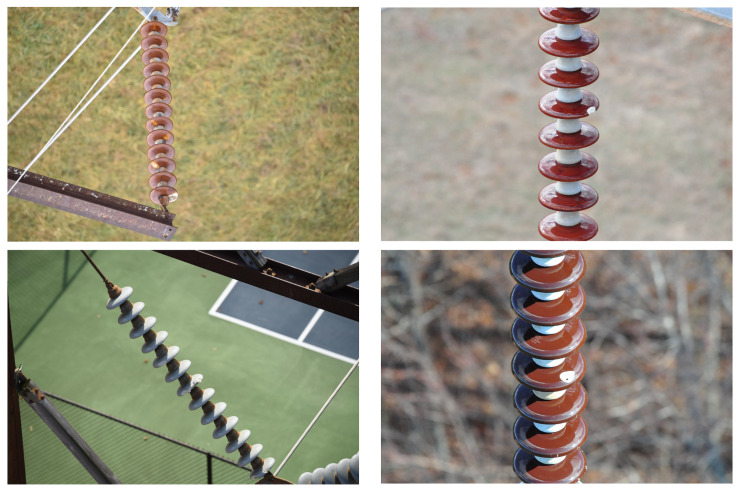
IDFF Dataset.

This IFDD dataset focuses on various types of insulator defect detection and contains nine label categories, namely, Glassdirty, Glassloss, Polymer, Polymerdirty, Two glass, Broken disc, Insulator, Pollution-flashover, and Snow. The dataset consists of 1607 images, which are split into a training set (1285 images), test set (162 images), and validation set (160 images) in an approximate 8:1:1 ratio. Representative samples are shown in Figure 7.

### 4.2. Quantitative Metrics

To comprehensively evaluate the performance of the proposed DB-YOLO model in insulator defect detection, multiple quantitative metrics are employed. Conventional studies often rely on Precision, Recall, and mean Average Precision (mAP), which provide useful but limited insights into model behavior. In this work, we extend the evaluation to include additional indicators, such as F1-Score, False Positive Rate (FPR), and True Negative Rate (TNR), so as to ensure a more rigorous and balanced assessment.

Precision is defined as the ratio of true positive predictions to the sum of true positives and false positives, which can be expressed as follows:

(5) $$\mathrm{Precision}=\frac{TP}{TP+FP}$$

where TP and FP denote true positives and false positives, respectively. Recall measures the ability of the model to detect actual defects and is given by the following:

(6) $$\mathrm{Recall}=\frac{TP}{TP+FN}$$

where FN represents false negatives. To balance Precision and Recall, the *F*1-Score is introduced as their harmonic mean, expressed as follows:

(7) $$F1=\frac{2\times \mathrm{Precision}\times \mathrm{Recall}}{\mathrm{Precision}+\mathrm{Recall}}$$

To further examine the diagnostic reliability in engineering applications, we introduce FPR and TNR. The False Positive Rate quantifies the probability of misclassifying non-defect samples as defects and is expressed as follows:

(8) $$\mathrm{FPR}=\frac{FP}{FP+TN}\;$$

while the True Negative Rate measures the proportion of correctly identified non-defect samples, expressed as follows:

(9) $$\mathrm{TNR}=\frac{TN}{TN+FP}$$

where TN denotes true negatives. Furthermore, this study adopts mAP@50 as the standard metric for defect detection evaluation, which corresponds to the mean of average precision across all classes at an IoU threshold of 0.5 as follows:

(10) $$mAP@50=\frac{1}{N}\sum_{i=1}^{N}AP_{i}^{\left(0.5\right)}\;$$

where $AP_{i}^{\left(0.5\right)}$ denotes the average precision of class *i* at IoU = 0.5, and *N* is the number of defect categories. It should be emphasized that all reported mAP values in this study refer to mAP@50. Inference speed is reported in terms of FPS, which allows the assessment of the model’s practical efficiency. A higher FPS indicates shorter processing time per image, providing a direct measure of suitability for real-time or near real-time engineering inspection tasks.

### 4.3. Model Training

The proposed framework is implemented in PyTorch 2.1 and executed on a workstation equipped with an NVIDIA RTX 3090 GPU. Training is performed in GPU mode with CUDA 11.3.1 and cuDNN 8.2.1. Model parameters are optimized using the stochastic gradient descent (SGD) algorithm, with the detailed training configurations summarized in Table 2.

### 4.4. Comparison with State-of-the-Art Methods

The experimental results on the China Power Line Insulator dataset are presented in Table 3. The proposed method achieves the highest mAP50 (99.6%) and an accuracy of 91.2% while maintaining a recall of 100%. The F1-score reaches 95.3%, with the FPR reduced to 4.0% and the TNR improved to 96.0%, indicating strong robustness.

In terms of efficiency, the proposed method reaches 60 FPS, corresponding to approximately 16.7 ms per image. Although this speed is lower than FCOS (125 FPS, 8.0 ms) and YOLOv4 (91 FPS, 11.0 ms), it still satisfies the near real-time requirements of UAV inspection. Compared with YOLOv11, the second-best model, our method improves mAP50 by 0.1%, accuracy by 4.4%, and F1-score by 2.3%. Relative to YOLOv5, the accuracy gain reaches 5.1%. These results demonstrate that the slight sacrifice in inference speed is compensated by substantial improvements in accuracy and robustness, which are more valuable for engineering applications.

On the IFDD dataset (Table 4), the proposed method achieves 92.2% accuracy, 89.5% recall, 93.1% mAP50, and 90.8% F1-score, with an FPR of 3.8% and a TNR of 96.2%, outperforming all comparison models. The inference speed is 58 FPS (17.2 ms per image), slightly slower than FCOS (121 FPS, 8.3 ms) and several YOLO variants, but still within a practical range for engineering deployment.

On the Yunnan Lijiang Power Line Insulator dataset (Table 5), the proposed method achieves the highest mAP50 (81.4%) across all categories and shows superior performance in multiple defect types, including cracks, contamination, rust, and missing caps. The inference speed reaches 78 FPS (12.8 ms per image), which is lower than FCOS (130 FPS, 7.7 ms) and some YOLO models, yet it remains sufficient for UAV inspection tasks.

**Figure 8:**
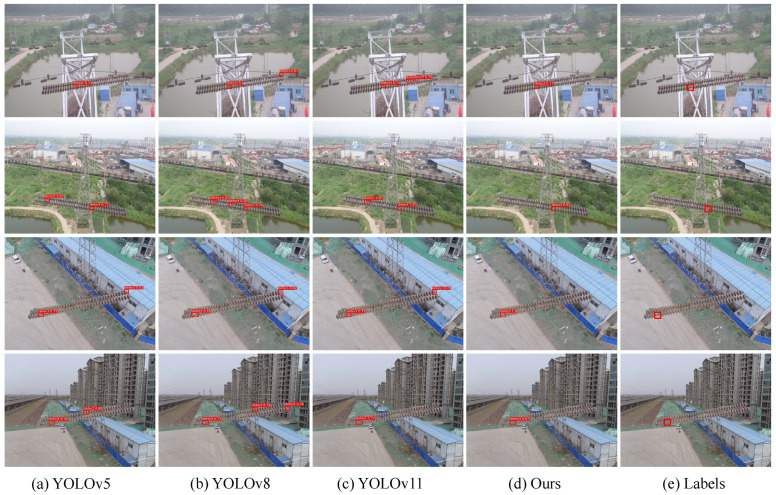
Results from the Chinese Power Line Insulator Dataset.

**Figure 9:**
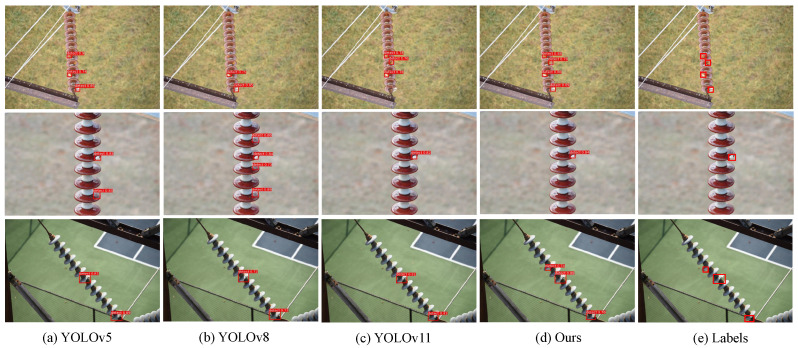
Result from the IDFF Dataset.

**Figure 10:**
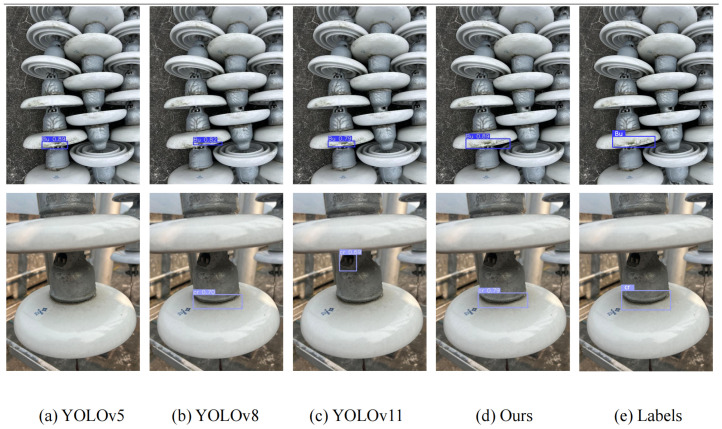
Result from the Yunnan Lijiang Power Line Insulator Dataset.

In addition to the quantitative results, qualitative comparisons are presented in Figure 8, Figure 9 and Figure 10. On the China Power Line Insulator dataset (Figure 8), the proposed method effectively identifies small-scale defects under complex backgrounds and significantly reduces false detections compared with baseline models. On the IFDD dataset (Figure 9), the proposed method demonstrates higher localization accuracy for small targets or partially occluded defects while maintaining robustness against background interference. On the Yunnan Lijiang dataset (Figure 10), the proposed method exhibits strong adaptability to various defect types and is capable of accurately detecting defects of different scales even in complex scenarios. These qualitative results further confirm that the proposed model not only surpasses comparison methods in numerical metrics but also provides reliable detection performance in real-world engineering environments.

Overall, the proposed method demonstrates clear advantages in accuracy and robustness while maintaining competitive diagnostic efficiency. Although it is not the fastest model, its balance between inference speed and detection performance makes it highly suitable for real-world engineering applications, ensuring reliability, safety, and operational efficiency.

### 4.5. Ablation Experiment

To evaluate the contributions of each module, we conducted ablation experiments on the following three datasets: CPLID, IFDD, and the Yunnan Lijiang Power Line Insulator Dataset, using YOLOv11 as the baseline. Table 6 presents the precision, recall, F1-score, and FPS for each module combination. Introducing the SHUFFLE module led to a notable increase in inference speed, with CPLID FPS rising from 62 to 66 (+4) and Lijiang dataset FPS from 80 to 84 (+4). However, the precision and F1-score slightly decreased, with CPLID F1 dropping from 93.0% to 91.8% (−1.2%), IFDD F1 from 89.9% to 88.8% (−1.1%), and Lijiang F1 from 78.4% to 77.1% (−1.3%). This indicates that while SHUFFLEBone improves computational efficiency, it introduces minor information loss, which is particularly evident in the CPLID dataset dominated by small targets.

After incorporating the EMB, the performance was substantially recovered and further enhanced. The F1-score increased to 93.4% for CPLID (+1.6% relative to SHUFFLEBone), 90.4% for IFDD (+1.6%), and 79.4% for Lijiang (+2.3%), demonstrating that EMB effectively enhances multi-scale feature representation and is especially crucial for small-target detection in CPLID. The improvements on the IFDD and Lijiang datasets further show that EMB also benefits medium- and large-target detection, confirming its key role in overall model accuracy.

Adding the Query Block (QB) module further improved detection performance, with F1-scores reaching 94.4% for CPLID (+1.0%), 90.6% for IFDD (+0.2%), and 80.5% for Lijiang (+1.1%). QB enhances feature selection and boundary localization, leading to more robust detection in complex backgrounds. Finally, integrating AWCIOU loss with the complete Mamba mechanism yielded F1-scores of 95.3% for CPLID (+0.9%), 90.8% for IFDD (+0.2%), and 81.2% for Lijiang (+0.7%), representing improvements of +2.3%, +0.9%, and +2.8% points relative to the baseline, respectively. These results highlight that EMB is the primary contributor for small-target performance in CPLID, while the combination of QB and AWCIOU ensures balanced gains for medium and large targets in the IFDD and Lijiang datasets.

Overall, the ablation study clearly quantifies the contributions of each module. SHUFFLE improves inference speed but slightly reduces F1-score; EMB is critical for enhancing small-target and multi-scale feature representation; and QB and AWCIOU further refine feature selection and localization, yielding consistent improvements across all datasets.

## 5. Discussion

### 5.1. Lightweight Comparison Experiment

We conducted comparative experiments using several mainstream lightweight backbones, including MobileNetV3 [49], GhostNet [50], and ShuffleNetV2 [51], as the convolutional modules for YOLOv11. A detailed comparison with the baseline YOLOv11s is presented in Table 7.

As shown in Table 7, ShuffleNetV2 (referred to as “shuffle”) achieved the highest F1-score of 76.2% among the evaluated lightweight networks and a competitive mAP50 of 77.4%, while significantly reducing computational cost, with only 2.45 G FLOPs and a model size of 2.3 M parameters. In comparison, YOLOv11s + MobileNetV3 obtained an F1-score of 70.5% and mAP50 of 72.1% with 3.3 G FLOPs and 7.3 M parameters, while YOLOv11s + GhostNet achieved 71.0% F1 and 72.7% mAP50 with 5.4 G FLOPs and 6.7 M parameters. These results demonstrate that ShuffleNetV2 not only maintains relatively high detection accuracy but also substantially improves efficiency in terms of FLOPs, model size, and computational cost, highlighting its excellent lightweight characteristics and validating the effectiveness of our proposed backbone modifications.

### 5.2. Curve-Based Comparison

**Figure 11:**
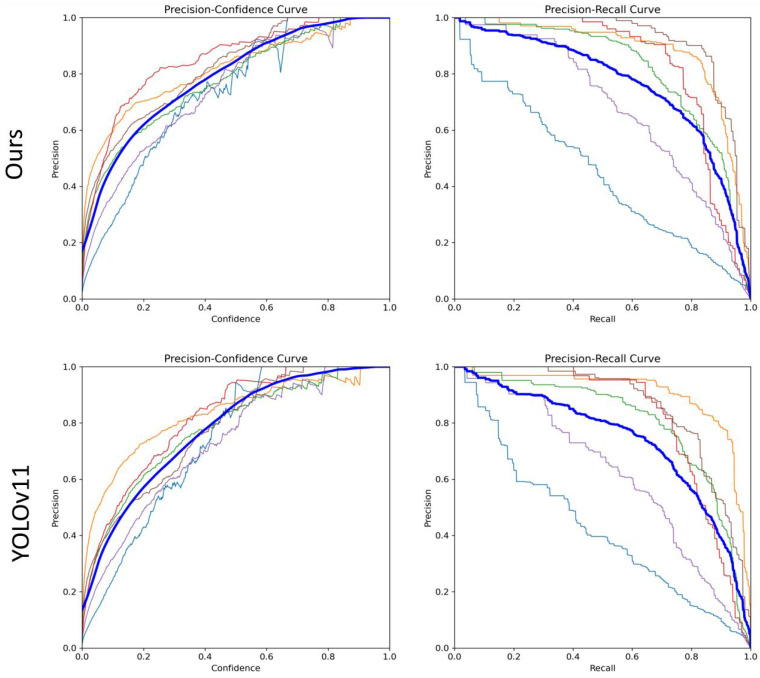
Model curves of different metrics. The blue curve represents the mean curve across all classes, while the other colored curves correspond to different individual classes.

To comprehensively evaluate the performance of our proposed method against the baseline YOLOv11, we conducted curve-based comparisons on the Yunnan Lijiang Power Line Insulator Dataset using Precision–Recall (PR) and Precision–Confidence (PC) curves (Figure 11). The blue curve represents the average performance across all defect categories, while the colored curves correspond to individual defect types. On the PR curve, our method consistently outperforms YOLOv11 across the full recall range. Notably, it maintains higher precision at high recall levels, demonstrating superior accuracy and robustness for multi-category defect detection, while still achieving high precision at low recall levels, indicating a low false positive rate.

On the PC curve, the proposed method shows a stabler and slower decline in precision at low confidence thresholds, whereas YOLOv11’s precision drops more rapidly, indicating less reliable predictions at lower confidence levels. This highlights the stronger confidence calibration of our model, which helps reduce false alarms in automated inspection scenarios.

Overall, the curve-based analysis demonstrates that the proposed method not only improves detection accuracy over YOLOv11 but also maintains reliable performance under high-recall and low-confidence conditions, illustrating its advantages for practical engineering applications.

### 5.3. Comparison with Multi-Branch YOLO Models

To further evaluate the effectiveness of the proposed dual-branch detection framework, we conducted a comparative study with several recently proposed multi-branch YOLO models, including UAV-YOLOv8, RDS-YOLO, and MAF-YOLO. These models are specifically designed to improve detection performance for objects of varying scales, particularly focusing on small targets. The comparison was performed on the following three widely used insulator defect datasets: CPLID, IFDD, and Lijiang. The evaluation metrics include Precision, Recall, model parameters (Params), computational complexity (FLOPs), and inference speed (FPS), providing a comprehensive assessment of both detection accuracy and efficiency. The results are summarized in Table 8.

The experimental results on the CPLID, IFDD, and Lijiang datasets show that on the CPLID dataset, our proposed method achieves the highest precision of 91.2% and maintains a recall of 100%, outperforming UAV-YOLOv8, RDS-YOLO, and MAF-YOLO. In addition, our model achieves the best trade-off between accuracy and efficiency, with only 7.1 M parameters and 6.8 G FLOPs, while running at 60 FPS. These results indicate that our dual-branch framework can effectively capture both large- and small-scale defect features without introducing excessive computational overhead.

For the IFDD dataset, our method again achieves the best overall performance, reaching 92.2% precision and 89.5% recall. Compared to UAV-YOLOv8, which requires more than 31 G FLOPs, our approach significantly reduces computational cost to 6.8 G while preserving competitive accuracy. Compared to lightweight models such as RDS-YOLO and MAF-YOLO, our framework demonstrates higher detection accuracy while maintaining similar real-time performance, confirming its superiority in balancing effectiveness and efficiency.

On the Lijiang dataset, which contains more challenging conditions with smaller defects and complex backgrounds, our approach consistently demonstrates some advantages. The proposed method achieves 82.3% precision and 80.1% recall, outperforming other multi-branch YOLO variants by a clear margin. In addition, the FPS of our method remains at 78, which is higher than UAV-YOLOv8 and comparable to other lightweight competitors, further validating its suitability for deployment in real-world UAV-based inspection tasks.

Overall, these results demonstrate that the proposed dual-branch framework effectively addresses the challenges of insulator defect detection across different datasets. By explicitly incorporating small-defect and large-defect detection branches, the model achieves superior accuracy across scales, while its optimized architecture ensures low computational complexity and real-time inference.

## 6. Conclusions

In this paper, we proposed a novel dual-branch YOLO algorithm based on YOLOv11 to address the challenge of detecting insulator defects of varying sizes in real-world power line inspection scenarios. By introducing a dual-branch structure that separately focuses on large and small defects, the proposed method ensures more accurate and effective detection across a wide range of defect scales. Additionally, the integration of the Mamba algorithm for enhanced global feature extraction, combined with an adaptive weighted CIoU loss function tailored to different defect sizes, further optimizes detection performance while reducing training complexity. To improve efficiency, we adopt the lightweight ShuffleNetV2 as the backbone network, enabling real-time inference without compromising accuracy.

Experimental results on three insulator-related datasets—the CPLID, IFDD, and Lijiang dataset—demonstrate that our approach achieves superior performance in both detection accuracy and processing speed. This makes it highly applicable to real-world inspection tasks such as UAV-based automatic insulator defect detection.

Although the proposed dual-branch YOLO algorithm shows strong performance in insulator defect detection, several limitations remain. First, while the adaptive weighted CIoU loss improves detection across different defect sizes, the model’s robustness may still be challenged in complex backgrounds such as occlusion by trees or interference from wires. Second, although we evaluated the model on multiple datasets, the current data still covers a limited range of scenarios. Future work could explore the expansion of dataset diversity—across various climates, viewing angles, and operational environments—to further enhance the model’s generalizability and robustness.

## Figures and Tables

**Table 1:** Summary of insulator defect detection datasets.

Dataset	Source	Train/Val/Test	Classes
CPLID	State Grid Corporation of China, Beijing, China	600/124/124	2
Lijiang	Yunnan Power Grid Co., Ltd., Kunming, China	2800/600/600	6
IFDD	InterNet, online, N/A	1285/160/162	9

**Table 2:** Experimental parameter settings.

Parameter	Numerical Value
Epochs	200
Batch size	16
Learning rate	0.01
Weight decay	0.0005
Momentum	0.937
Image size	640 × 640

**Table 3:** Comparison of different methods on the Chinese Power Line Insulator Dataset.

Model (%)	Precision (%)	Recall (%)	mAP50 (%)	F1-Score (%)	FPR (%)	TNR (%)	FPS
FCOS [35]	81.0%	100%	99.4%	89.5%	9.7%	90.3%	125
YOLOv4 [36]	84.9%	100%	99.5%	91.8%	7.3%	92.7%	91
YOLOv5 [37]	86.1%	100%	99.5%	92.3%	6.7%	93.3%	71
YOLOv8 [38]	86.2%	100%	99.5%	92.3%	6.7%	93.3%	62
YOLOv9 [39]	84.0%	99.2%	99.4%	91.0%	7.8%	92.2%	62
YOLOv11 [40]	86.8%	100%	99.5%	93.0%	6.3%	93.7%	62
Ours	91.2%	100%	99.6%	95.3%	4.0%	96.0%	60

**Table 4:** Comparison of different methods on the IFDD Dataset.

Model (%)	Precision (%)	Recall (%)	mAP50 (%)	F1-Score (%)	FPR (%)	TNR (%)	FPS
FCOS [35]	85.5%	83.1%	88.7%	84.3%	7.1%	92.9%	121
YOLOv4 [36]	88.5%	88.3%	91.9%	88.4%	5.8%	94.2%	95
YOLOv5 [37]	87.7%	84.1%	89.4%	85.9%	5.9%	94.1%	73
YOLOv8 [38]	90.4%	91.5%	91.6%	90.9%	4.9%	95.1%	65
YOLOv9 [39]	90.3%	87.0%	91.7%	88.6%	4.7%	95.3%	67
YOLOv11 [40]	91.1%	88.8%	92.2%	89.9%	4.4%	95.6%	63
Ours	92.2%	89.5%	93.1%	90.8%	3.8%	96.2%	58

**Table 5:** Comparison of different methods and their performance on the Yunnan Lijiang Power Line Insulator Dataset.

Model (%)	Precision (%)	Recall (%)	mAP50 (%)	F1-Score (%)	FPR (%)	TNR (%)	FPS
Faster RCNN [41]	75.3%	72.6%	74.1%	73.9%	8.0%	92.0%	55
Cascade RCNN [42]	74.2%	71.5%	73.3%	72.8%	8.2%	91.8%	52
Libra RCNN [43]	78.1%	76.4%	77.1%	77.2%	7.1%	92.9%	64
TridentNet [44]	77.5%	76.2%	77.0%	76.8%	7.6%	92.4%	73
YOLOv3 [45]	72.4%	70.8%	72.0%	71.6%	9.1%	90.9%	90
RetinaNet [46]	69.3%	66.5%	68.4%	67.9%	9.8%	90.2%	91
FCOS [35]	74.6%	73.2%	73.9%	73.9%	8.7%	91.3%	130
YOLOv7 [47]	75.8%	73.6%	74.2%	74.7%	8.2%	91.8%	84
YOLOv8 [38]	76.4%	74.7%	75.2%	75.5%	7.8%	92.2%	85
EDDN [48]	73.5%	71.3%	72.4%	72.4%	8.7%	91.3%	81
YOLOv11 [40]	79.6%	77.2%	78.5%	78.4%	6.9%	93.1%	80
Ours	82.3%	80.1%	81.4%	81.2%	5.8%	94.2%	78

**Table 6:** Ablation study on the three datasets.

Components	CPLID				IFDD				Lijiang			
	Prec. (%)	Rec. (%)	F1 (%)	FPS	Prec. (%)	Rec. (%)	F1 (%)	FPS	Prec. (%)	Rec. (%)	F1 (%)	FPS
Baseline	86.8%	100%	93.0%	62	91.1%	88.8%	89.9%	63	79.6%	77.2%	78.4%	80
+ SHUFFLE	85.2%	99.2%	91.8%	66	89.7%	87.9%	88.8%	68	78.2%	76.1%	77.1%	84
+ EMB	87.5%	100%	93.4%	64	91.8%	89.1%	90.4%	62	80.4%	78.5%	79.4%	81
+ QB	89.4%	100%	94.4%	62	92.0%	89.3%	90.6%	60	81.5%	79.6%	80.5%	80
+ AWCIOU	91.2%	100%	95.3%	60	92.2%	89.5%	90.8%	58	82.3%	80.1%	81.2%	78

**Notes:** SHUFFLE refers to Shufflebone, Prec. stands for Precision, Rec. stands for recall, and F1 stands for F1-score.

**Table 7:** Effectiveness comparison of Shufflebone with different backbones.

Model	mAP50 (%)	F1-Score (%)	FLOPs (G)	Parameters (M)
YOLOv11s	79.6	78.4	6.5	9.4
YOLOv11s + Mobilenet V3	72.1	70.5	3.3	7.3
YOLOv11s + GhostNet	72.7	71.0	5.4	6.7
YOLOv11s + ShuffleNet V2	77.4	76.2	2.45	2.3

**Table 8:** Performance comparison of different algorithms on the three datasets.

Dataset	Algorithm	Precision (%)	Recall (%)	Params (M)	FLOPs (G)	FPS
CPLID	UAV-YOLOv8	88.5	100	10.3	31.4	47
	RDS-YOLO	85.0	100	2.7	10.3	59
	MAF-YOLO	87.2	99.2	3.8	10.5	55
	**Ours**	**91.2**	**100**	**7.1**	**6.8**	**60**
IFDD	UAV-YOLOv8	90.0	87.0	10.3	31.4	44
	RDS-YOLO	88.3	85.5	2.7	10.3	58
	MAF-YOLO	89.1	86.0	3.9	11.7	54
	**Ours**	**92.2**	**89.5**	**7.1**	**6.8**	**58**
Lijiang	UAV-YOLOv8	79.5	77.0	10.3	31.4	72
	RDS-YOLO	80.0	78.5	2.7	10.3	77
	MAF-YOLO	81.0	79.0	3.8	10.5	77
	**Ours**	**82.3**	**80.1**	**7.1**	**6.8**	**78**

## Data Availability

All data supporting the reported results in this study are available from the corresponding author upon reasonable request, due to privacy and ethical restrictions.
